# Dr. Thomas Whitehead Reid

**Published:** 1910-04-09

**Authors:** 


The death has taken place at Canterbury of Dr. Thomas Whitehead Reid. He studied at St. Bartholomew's Hospital, took his M.R.C.S. in 1874, and became an M.D. of St. Andrews University and Fellow of the R.C.P. of Edinburgh. He held, however, many surgical appointments, and was formerly ophthalmic house surgeon and registrar at St. Bartholomew's Hospital, and later senior surgeon to the Kent and Canterbury Hospital. Dr. Reid was a member of the Central Council and chairman of the Hospitals Committee of the British Medical Association, a member of the London Medical Society, and a Fellow of the Royal Society of Medicine.

